# BMAL1 in Ischemic Heart Disease: A Narrative Review from Molecular Clock to Myocardial Pathology

**DOI:** 10.3390/ijms26104626

**Published:** 2025-05-12

**Authors:** Jingyi Yang, Junxin Zhao, Zhuoyang Chen, Lincheng Duan, Hong Yang, Dingjun Cai

**Affiliations:** 1 Acupuncture and Tuina School, Chengdu University of Traditional Chinese Medicine, Chengdu 611137, China; yangjingyi@stu.cdutcm.edu.cn (J.Y.); xiaoyuzhao631@gmail.com (J.Z.); chenzhuoyang2025@163.com (Z.C.); lincheng@stu.cdutcm.edu.cn (L.D.); yanghong95ob@163.com (H.Y.); 2 Key Laboratory of Acupuncture for Senile Disease (Chengdu University of TCM), Ministry of Education/Acupuncture and Chronobiology Key Laboratory of Sichuan Province, Chengdu 611137, China

**Keywords:** BMAL1, myocardial ischemia, time rhythm

## Abstract

The biological clock is crucial for controlling the circadian rhythm of the human body and maintaining the stable cyclic changes of various human life activities. Cardiovascular disease has become one of the primary problems affecting human life and health in today’s society. Cardiovascular disease exhibits distinct circadian rhythms, with the core clock gene protein Brain and muscle ARNT-like protein 1 (BMAL1) playing critical roles in both physiological cardiac function and pathological processes. BMAL1 regulates myocardial gene expression, maintains normal structures, and stabilizes circadian rhythms to preserve cardiac homeostasis. In the pathological state of myocardial ischemia, BMAL1 ameliorates myocardial ischemic injury by regulating intrinsic mechanisms such as oxidative stress response, energy metabolism, immune-inflammatory response, and apoptosis and autophagy in cardiomyocytes. This review systematically examines BMAL1’s involvement in myocardial ischemic injury through the circadian regulation of cardiac function. We analyze its multidimensional impacts on oxidative stress, energy metabolism, immune-inflammatory responses, apoptosis, and autophagy, highlighting the biological significance of this clock gene in ischemic pathophysiology.

## 1. Introduction

Cardiovascular disease (CVD) is becoming more prevalent globally each year, posing a significant health risk. Coronary Artery Disease (CAD) primarily manifests as acute myocardial infarction and unstable angina, with atherosclerosis serving as its pathophysiological cornerstone. This process originates from endothelial dysfunction [1] and inflammation [2], progressing through the development of stable or vulnerable plaques [3], ultimately leading to vascular stenosis, plaque rupture, and thrombus formation that culminate in myocardial ischemia and infarction [4]. The diagnosis of CAD requires a combination of clinical presentation combined with high-sensitivity troponin (hs-cTn) and electrocardiography to detect acute ischemia. Non-invasive investigations such as coronary CT angiography (CCTA) and myocardial perfusion imaging can assist in the assessment, while invasive angiography combined with fractional flow reserve (FFR) and optical coherence tomography (OCT) can be used to accurately analyze the nature of the plaque and the risk of ischemia [5]. With the deepening of the understanding of disease diagnosis, the diagnosis of coronary heart disease should be centered on CCTA, integrating plaque morphology, non-invasive functional assessments (FFR-CT), and stratified diagnostic and treatment strategies and promoting the diagnosis of coronary heart disease from “symptom-driven” to “risk-driven” [6]. According to WHO, CVD-related deaths could reach 23 million by 2030 [7]. Ischemic heart disease (IHD) has caused 9.14 million deaths since 1990. By 2019, the global count of individuals with IHD rose to 197 million [8]. The healthcare burden from its high mortality and disability rates has been on the rise [9]. Cardiovascular disease has become one of the primary problems affecting human life and health in today’s society, and how to prevent and control ischemic heart disease has been the focus of clinical research.

Brain and muscle ARNT-like protein 1 (BMAL1) is a key circadian rhythm transcription factor. Through in-depth analysis of the protein structure and function of BMAL1, scientists have found that its protein structure contains multiple phosphorylation and acetylation sites, and its interacting proteins are intimately connected to the emergence and advancement of cardiovascular diseases in the clinic; therefore, it has an important potential value in cardiovascular disease study and therapy [10]. BMAL1, a central component of the biological clock, binds to the clock rhythm regulator molecule (circadian locomotor output cycles kaput (CLOCK)) to constitute a heterodimer [11]. This compound is able to interact directly with the E-Box element to activate the key genes of the periodic circadian rhythm, period circadian regulator (PER), and cryptochrome circadian regulator (CRY) [12]. Accordingly, accumulation of the PER and CRY protein complexes represses BMAL1/CLOCK expression, forming a negative feedback loop to maintain the dynamic stability of circadian rhythms [11].

Multiple physiological activities of the heart exhibit distinct circadian rhythms, and people with chronic day and night upside-down jobs are at a significantly increased risk for cardiovascular disease [13]. In particular, events such as myocardial ischemia, sudden cardiac death, and myocardial infarction are more likely to happen in the morning [14]. These phenomena suggest that disruption of biological rhythms may be an important risk factor for the development of cardiovascular disease. This phenomenon may be related to the central biological clock in the suprachiasmatic nucleus (SCN) of the hypothalamus, which acts as a central rhythmic pacemaker and influences the peripheral biological clock system and electrophysiological properties of the heart through various neurohumoral pathways [15,16]. Despite evidence linking circadian disruption to cardiovascular risk, the molecular pathways connecting peripheral cardiac clocks to disease progression remain poorly understood. This provides a chronomedicine perspective for clinical practice, highlighting the necessity to incorporate circadian rhythms into cardiovascular disease prevention, diagnosis, and therapeutic strategies.

Although the specific mechanism of myocardial ischemia-induced myocardial injury has not been fully clarified, research has shown that BMAL1 is crucial in regulating circadian rhythms, cell death, inflammatory responses, energy metabolism, oxidative stress, and gene expression, which influences the onset and development of myocardial ischemic injury. Therefore, the objective of this paper is to review the regulatory mechanisms of BMAL1 in myocardial ischemic injury and its potential applications for preventing and treating cardiovascular diseases.

## 2. Literature Search Strategy

The literature search for this review encompassed comprehensive coverage of international authoritative databases, including PubMed, Web of Science, and Embase, as well as Chinese databases, such as China National Knowledge Infrastructure (CNKI), Wanfang, and China Science and Technology Journal Database (VIP), to ensure a thorough retrieval of both English and Chinese publications. The search strategy was developed using the core terms “BMAL1” and “ischemic heart disease” derived from the Medical Subject Headings (MeSHs) thesaurus, with no time restrictions applied to fully integrate historical foundations and recent advancements in the field. The literature screening prioritized thematic relevance, emphasizing the inclusion of highly cited original studies, reviews, and methodological papers. Manual searches of high-impact journals within the field were additionally performed. Although this narrative review did not adhere to the standardized search protocols of systematic reviews, cross-verification across multiple databases and iterative keyword optimization ensured the representativeness of the selected literature in elucidating the mechanistic links between BMAL1 and ischemic heart disease. This approach established a robust evidence base to support subsequent theoretical explorations.

## 3. Cardiac Regulation by BMAL1

### 3.1. Regulation of the Cardiac Vasculature by BMAL1

Vascular lesions are important risk factors for myocardial ischemia, including coronary artery atherosclerosis and vascular endothelial injury, all of which cause endothelial and microvascular damage and affect neovascularization in coronary arteries. Vascular lesions are critical triggers for thrombosis. Thrombosis can exacerbate the narrowing of the coronary arteries, and, more seriously, dislodged blood clots can cause critical illnesses, such as acute myocardial infarction. In a mouse model, BMAL1/CLOCK modulates the response to stimulated thrombus (thrombotic vascular occlusion after photochemical injury, TTVO) formation in vivo. This phenomenon may interact with the environmental variable light, and BMAL1 deficiency leads to a disruption of the organism’s circadian rhythm and increases the organism’s susceptibility to photochemical stimuli, which may contribute to cardiovascular events [17]. Leukocyte recruitment in arteries and veins of the large and microvessels in mice also has a circadian rhythm. The expression of BMAL1 in leukocytes, endothelial cells, and arterial wall cells maintains rhythmic leukocyte adhesion in arteries and veins, and its absence disrupts this rhythmicity, which, in turn, leads to altered rhythmicity of the inflammatory response and thrombosis, increasing the risk of acute cardiovascular complications [18]. In addition to the effects on thrombosis, endogenous molecular biological clocks have been shown to exist in vascular endothelial and peripheral cells [19]. Studies have shown that *BMAL1 knockout mice* develop vascular endothelial injury and a specific deletion of bone marrow-derived progenitor cells, which is manifested by aberrant vascular remodeling, increased susceptibility to large vessel thrombosis, and microvascular injury, which is closely associated with superoxide radicals, enhanced inflammatory responses, and reduced atheromatous plaque stability or rupture [20,21]. Unstable atherosclerotic plaque is also a potential risk factor for ischemic heart disease. Research in mice indicated that BMAL1 could facilitate the transformation of vascular smooth muscle cells (VSMCs) to fibroblasts in stable atherosclerotic plaques by upregulating Yes-associated protein 1 (YAP1) expression. Additionally, BMAL1 can inhibit the extent of atherosclerotic plaque lesions and down-regulate the levels of Matrix Metalloproteinase-2 (MMP-2) and Matrix Metalloproteinase-9 (MMP-9) in atherosclerotic plaques, exerting an anti-atherosclerotic effect and reducing the incidence of ischemic heart disease [22,23]. At the microvascular level, BMAL1 plays an opposite role. After myocardial infarction, the myogenic response of resistance arteries in skeletal muscle tissue (mediated by vascular smooth muscle) becomes heightened, leading to increased microvascular constriction and exacerbating post-MI ischemic injury. Notably, this myogenic response exhibits circadian rhythmicity. Disruption of the circadian clock abolishes the MI-induced enhancement of the myogenic response. Consequently, reduced myogenic tone and total peripheral resistance (TPR) in *BMAL1-knockout mice* improve cardiac function and attenuate infarct expansion [24].

In summary, BMAL1 plays a crucial role in maintaining vascular homeostasis, reducing thrombosis, stabilizing atherosclerotic plaques and microvascular injury, and regulating biorhythms. BMAL1 expression could be an aim for preventing and improving the prognosis of ischemic heart disease.

### 3.2. BMAL1 Regulation of Cardiac Function Circadian Rhythm

BMAL1 is a core clock gene in the heart, and deletion or mutation of BMAL1 is closely associated with rhythmic alterations in the heart, with important effects on several aspects, including genes, blood pressure, myocardial electrical activity, and cardiac systolic and diastolic function.

Circadian rhythms are a natural internal process of living organisms, which can adjust any oscillating biological process with a period of about 24 h. The 24-hour rhythms are driven by natural circadian rhythms, and the biological clock within organisms allows them to adapt consistently and accurately to changes in the environment, including light, temperature, and food. The system of clocks comprises a central circadian clock in the hypothalamus’s supraoptic nucleus and peripheral circadian clocks in other brain regions and body tissues, including muscles, adipose tissue, and the liver [15]. About 8% to 10% of the genes in the heart are regulated by biological clock genes that are present in cardiomyocytes and display rhythmicity [25,26]. The circadian biological clock is regulated by two interlocking transcription/translation feedback loops (TTFL), and its core clock genes consist of four proteins: BMAL1, CLOCK, PER, and CRY. CLOCK and BMAL1 constitute a heterodimeric complex, which activates PER and CRY and their downstream gene transcription by regulating the interaction of gene E-Box elements. The PER/CRY protein complex accumulates in the nucleus and, in turn, restrains the transcriptional activity of BMAL1/CLOCK, constituting a negative feedback loop that creates a circadian oscillation of approximately 24 h. The nuclear receptor subfamily (REV-ERBs) and the retinoic acid receptor-related orphan receptor (ROR) are initiated by the heterodimerization of BMAL1/CLOCK to form a second feedback loop, in which REV-ERBs exert inhibitory effects on BMAL1, whereas RORs positively regulate BMAL1 expression by activating the BMAL1 gene promoter, conferring rhythmic stability [27,28,29,30].

A genome-wide analysis of single nucleotide polymorphisms in the human population revealed that *BMAL1* was significantly associated with coronary artery disease [31]. Genetic polymorphisms in *BMAL1* are possible risk factors for myocardial infarction [32]. Correlation studies of genotype–phenotype interactions have also shown that *BMAL1* genetic mutations are associated with circadian rhythm phenotypes in patients with myocardial infarction [33]. The measurement of *BMAL1* mRNA in cardiac tissue showed a clear circadian rhythm in the human heart, with peaks in *PER* and troughs in *BMAL1* coinciding with times of day when cardiovascular events are high [34]. Additionally, it has also been found that there are substantial disparities in circadian rhythm genes of cardiovascular disease between physiological sexes. For example, BMAL1 was more sensitive to methamphetamine in ischemic myocardium of female rats compared to male rats, whereas this phenomenon was not found in female rats, so there may be sex differences in the regulation of myocardial ischemia by BMAL1 [35]. Gene-level studies have revealed an alteration in *BMAL1* at the gene level in the pathological state of myocardial ischemia, providing new ideas for the in-depth study of *BMAL1*. *BMAL1*, as an important core gene in the negative feedback loop, is placed upstream of the biological clock genes and is directly tightly associated with the progression of cardiac diseases, and its deletion can result in permanent loss of cardiac circadian rhythms [36].

Blood pressure exhibits a circadian rhythm over a 24-hour period and peaks in the morning [37]. BMAL1 protein levels in the normal rat heart show circadian oscillations, whereas BMAL1 protein expression in the myocardium of hypertensive rats loses its original volatility and rhythmicity [38]. BMAL1 deficiency also increases the rate of clinical cardiovascular events due to both the disturbance of circadian rhythms and the body’s decreased ability to handle increased blood pressure from environmental stressors [37]. In addition to its influences on the circadian rhythm of blood pressure, BMAL1 also affects myocardial electrical activity. Mice with a targeted knockout of *BMAL1* in cardiomyocytes develop a prolonged QT interval, slower repolarization, and a lower incidence of action potential alternans, leading to a greater susceptibility to arrhythmias [39]. In animal models, the rhythm disturbances of BMAL1 increase atrial HF events by affecting ion channels and calcium homeostasis, and the deficiency of BMAL1 leads to a decreased ability to inhibit fibrosis and oxidative stress, which causes cardiac fibrosis, stiffness, and enlargement, both of which combine to contribute to the onset and progression of atrial fibrillation (AF) [40]. Ventricular arrhythmia (VA) is one of the primary reasons for sudden cardiac death as a result of myocardial infarction. Animal studies show that BMAL1 can affect the occurrence of VAs by regulating the function of cardiomyocyte ion channels and the circadian rhythm of Ryanodine Receptor 2 (RYR2) [41]. In a rat myocardial infarction model, norepinephrine and isoprenaline show circadian rhythms in cardiomyocytes, and the CLOCK/BMAL1 dimer binds to the enhancer of the β1-adrenoceptor gene to upregulate its expression, thereby regulating the circadian rhythm of ventricular arrhythmias in rats with chronic heart failure. In summary, circadian rhythm oscillations of BMAL1 were significantly enhanced in rats with acute myocardial infarction, and its mediated β3-AR activation decreased the frequency of ventricular tachycardias (VTs) and ventricular fibrillations (VFs) with acute myocardial infarctions or healed myocardial infarctions, reducing the occurrence of adverse cardiac events [42].

Animal experiments show that *BMAL1* knockout also triggers age-related dilated cardiomyopathy, as well as myocardial diastolic dysfunction, sarcoplasmic nodal destruction, inflammatory responses, and other pathologic changes [43,44]. In addition, in cardiomyocyte-specific *BMAL1 knockout mice*, cardiomyocyte growth and metabolism are affected due to increased cardiac sensitivity to insulin-like growth factor 1 (IGF1) and growth hormones (GHs), resulting in cardiac remodeling, contractile dysfunction, cardiomyocyte hypertrophy, and cardiac fibrosis [45]. This cardiac fibrosis is also associated with the high expression of pro-inflammatory genes in mice due to the disruption of neutrophil chemotaxis, leukocyte migration, and monocyte/macrophage circadian rhythms following BMAL1 deletion [43]. In addition, restoration of BMAL1 circadian rhythm under pharmacological intervention attenuates cardiac fibrosis in myocardial infarction mice by restraining the AKT Serine/Threonine Kinase (AKT) signaling pathway and attenuating cardiac fibroblast (CF) proliferation and collagen production [46].

It can be seen that BMAL1 maintains the normal structural function and circadian rhythm oscillations of the heart through multiple pathways, and the rhythm disturbances triggered by its abnormal expression have a negative impact on cardiac physiopathological changes, such as genes, blood pressure, myocardial electrical activity, cardiac systolic and diastolic function, cardiac structure, myocardial fibrosis, and dilated cardiomyopathy.

## 4. Regulatory Mechanisms of Myocardial Ischemia by BMAL1

### 4.1. BMAL1 Is Involved in the Regulation of Oxidative Stress Responses

Oxidative stress is a state of imbalance between the oxidative and antioxidant systems in the body, resulting from an imbalance between the production of reactive oxygen species (ROS) and the capacity of the body’s endogenous antioxidant system to scavenge ROS. Excess ROS oxidize lipids, proteins, and nucleic acids, resulting in irreversible damage to cell membranes, DNA, and other cellular structures. Oxidative stress is crucial in the pathogenesis of hypoxia, cardiotoxicity, and ischemia/reperfusion-related cardiovascular diseases [47]. ROS and many antioxidant genes exhibit cyclic oscillations regulated by the biological clock genes. In the mouse model, the heterodimer formation of BMAL1 with CLOCK regulates the expression of genes related to reactive oxygen species generation [48]. The generation of reactive oxygen species in mitochondria exerts cardioprotective effects by mediating the regulation of multiple signaling pathways and transcriptional circuits under hypoxia, enhancing energy production and reducing cell death [44]. However, excessive ROS can cause irreversible damage to cardiomyocytes. Studies have shown that premature senescence and decreased cardiac function in *BMAL1-deficient mice* may be related to excessive ROS, and antioxidant therapy may protect cardiac telomeres from oxidation by partially scavenging ROS and normalize cardiac function in *BMAL1 knockout mice* [49]. Additionally, the oxidative stress state of the body causes damage to VSMC, which is the main cell that constitutes the vascular wall, regulates blood pressure, and maintains cardiovascular homeostasis by contracting and altering vascular diameter and tone. Structural and functional abnormalities are among the risk factors for cardiovascular disease. *BMAL1-deficient mice* aggravate atherosclerosis by promoting VSMC and monocyte migration, elevating reactive oxygen species levels, and impairing antioxidant function [50]. Coincidentally, this phenomenon was also observed in human aortic endothelial cells with BMAL1 down-regulation. Increased cellular ROS content and monocyte recruitment induced oxidative stress, inflammatory responses, and rhythmic disturbances, accelerating the process of atherosclerosis [51], and thrombosis triggered by the rupture of atherosclerotic plaques is an important risk factor for the development of ischemic heart disease. Nuclear factor erythroid 2-related factor 2 (NRF2) is an important transcription factor in the antioxidant-reducing system of organisms, which can activate downstream antioxidant genes to reduce ROS levels and maintain cellular homeostasis. Under oxidative stress, ROS generation is increased in VSMC, and BMAL1 can maintain the antioxidant function and inhibit apoptosis in VSMC by activating NRF2 and BCL-2 transcription [52,53,54]. In addition, in the rat model of ischemia–reperfusion, BMAL1 can positively regulate the NRF2/HO-1(Heme Oxygenase-1) signaling pathway, which could be associated with reduced ROS content [55]. Thus, BMAL1 is closely tied to the transcription and translation of reactive oxygen species; the inhibition of the NRF2 signaling pathway increased the reactive oxygen species, and VSMC injury caused by decreased BMAL1 may be the key factor in the happening of oxidative stress in the ischemic myocardium.

### 4.2. BMAL1 Is Implicated in the Regulation of Energy Metabolism

Biological clocks are major regulators of metabolism and physiological health. The circadian rhythm disruption caused by mutations in their genes causes metabolic abnormalities, such as hyperlipidemia, hyperglycemia, hypoinsulinemia, and glucose intolerance, in humans and mice. Metabolic disorders significantly increase the risk of developing cardiovascular disease [56,57]. Mice with BMAL1 deletion suffer from energy metabolism disorders, including obesity, hyperlipidemia, hyperglycemia, and hypoglycemia, which result in endothelial damage and dysfunction and, consequently, trigger the formation of cardiovascular disease [57].

Mitochondria are the main site of intracellular energy metabolism and deliver Adenosine Triphosphate (ATP) to most eukaryotic cells via oxidative phosphorylation (OXPHOS) [58]. Under persistent hypoxia, ATP produced by aerobic respiration and glycolysis is rapidly consumed, and the lack of ATP leads to impaired cardiac myocyte dynamics and an imbalance in ion transport, which can lead to diastolic dysfunction and cell rupture. A normal supply of ATP is important for maintaining the energy metabolism in the heart [59]. BMAL1 regulates ATP production by affecting mitochondrial function, which, in turn, affects the development of myocardial ischemia. Transcriptional-level studies revealed that the expression of mitochondrial fusion-related genes was downregulated in cardiac tissues of cardiac-specific *BMAL1 knockout mice.* This might result from diminished BMAL1 levels, which inhibited the adenosine 5-monophosphate-activated protein kinase (AMPK)-dependent expression of Silent Information Regulator 1(SIRT1)and the mitochondrial biogenesis marker peroxisome proliferator-activated receptor gamma coactivation factor 1α (PGC1α) activity. Diminished expression of mitochondrial fusion-related genes causes mitochondrial dysfunction, such as reduced number, altered morphology, impaired phagocytosis, and gathering of reactive oxygen species, which, in turn, triggers defective mitochondrial ultrastructure and impaired cardiac function. Under hypoxic conditions, the CLOCK/BMAL1 interactions of mice may play a protective role in the ischemic myocardium by inhibiting ROS production, decreasing the levels of mitochondrial fission, improving the ability of the mitochondrial electron transport chain to metabolize, and increasing the efficiency of ATP synthesis to preserve the cell’s regular energy metabolism and reduce cell death and myocardial systolic-diastolic dysfunction [60,61,62,63,64].

After 10–20 s of coronary ischemia, the myocardium relies on anaerobic glycolysis to provide energy, and if anaerobic glycolysis is limited, the heart will experience contracture stiffness and increased infarct size when the myocardial energy supply is cut off. Under myocardial ischemia, *PER2 knockout mice* have a dramatically increased infarct size due to impaired glycolysis and depletion of glycogen stores; thus, PER2 can regulate fatty acid metabolism in the ischemic myocardium. The heterodimer formed by CLOCK/BMAL1 binds to the E-Box element of PER1–3 and activates the gene transcription and translation to produce the PER proteins. BMAL1 may regulate myocardial fatty acid metabolism and reduce the myocardial infarction area by regulating PER2 gene expression [65,66].

An analysis of mice cardiac combinatorial microarray data identified 19 direct target genes of BMAL1, among them, nicotinamide phosphoribosyltransferase (NAMPT)-mediated nicotinamide adenine dinucleotide (NAD) metabolism with a circadian rhythm [67]. NAMPT is the key rate-limiting enzyme for NAD synthesis, and, as a key cofactor in energy metabolism, NAD deficiency leads to energy metabolism stagnation. Additionally, Krüppel-like factor 15 (KLF15), which is a downstream transcription factor regulated by BMAL1/CLOCK, has the ability to directly influence NAMPT [68]. Mice with a cardiomyocyte-specific knockout of KLF15 experience disrupted circadian rhythm of NAMPT in their cardiac tissues [69]. BMAL1/CLOCK, via Nuclear Receptor Subfamily 1 Group D Member 1/2 (NR1D1/NR1D2, REV-ERBα/β), also downregulates the clock-control genes E4 promoter-binding protein 4 (E4BP4), and E4BP4 expression hinders NAMPT expression. Simply stated, during the sleep phase, the BMAL1/CLOCK heterodimer stimulates NAMPT production through KLF15, while during the wakefulness phase, it inhibits NAMPT production through E4BP4 [70]. The circadian rhythm of NAMPT regulates the normal level of cardiac NAD, which ensures the normal energy metabolism of the heart. The knockout of BMAL1 results in the restriction of the synthesis of NAMPT/NAD and the abnormal energy metabolism of the heart. The p85α subunit of encoding phosphatidylinositol 3-kinase (PIK3R1), another target gene of BMAL1, also displays a role in circadian rhythm. It is a part of the insulin signaling cascade, and its downstream components, AKT and Glycogen Synthase Kinase 3 Beta (GSK3β), are influenced by the biological clock genes of cardiomyocytes, which affect mouse cardiac metabolism and contractile function [67].

It can be seen that impaired energy metabolism in cardiomyocytes seriously affects cardiac functional activities, and BMAL1 can play a role in maintaining normal cardiac metabolism by regulating mitochondrial function, affecting ATP production, regulating myocardial fatty acid metabolism, and regulating target genes, such as NAMPT/NAD, PIK3R1-p85α subunits, etc.

### 4.3. BMAL1 Contributes to the Modulation of Immune-Inflammatory Responses

Recent findings suggest that immune cell-mediated inflammatory responses play a key role in the progression and prognosis of ischemic heart disease [71]. Myocardial local and systemic inflammatory responses can trigger pathological changes such as apoptosis, oxidative stress, myocardial fibrosis, and rupture of atherosclerosis-prone plaques, which are important triggers for adverse cardiac remodeling after myocardial ischemia. Considering the distinct rhythmic nature of cardiovascular diseases, biological clock genes might affect the onset and outcome of myocardial ischemia by altering the immune-inflammatory response.

Animal experiments show that neutrophils have a circadian rhythm and that their infiltration promotes late remodeling after myocardial infarction. Circadian oscillations of neutrophils are influenced by BMAL1. After myocardial infarction, the expression of the BMAL1 is disordered, and the circadian oscillation of neutrophils is destroyed. BMAL1 may ameliorate the adverse consequences of myocardial infarction by affecting the aging and cardiac remodeling processes of neutrophils [72]. In addition, *BMAL1 knockout mice* showed elevated neutrophil chemotaxis, elevated transcript levels of leukocyte migration genes, and disrupted circadian rhythms in monocytes/macrophages, as well as high expression of pro-inflammatory genes in cardiomyocytes, leading to inflammation in the heart and fibrosis in tissues [43]. Nuclear Factor Kappa-Light-Chain-Enhancer of Activated B Cells (NF-κB) is a key molecule in this inflammatory signaling pathway, and the biological clock has been demonstrated to regulate NF-κB [73]. BMAL1 transcription is inhibited by the TLRs-NF-κB signaling axis, which enlists DNA Methyltransferase 1 (DNMT-1) to methylate the BMAL1 promoter. Down-regulation of BMAL1 further enhanced the NF-κB signal transduction and enhanced oxidative stress and inflammatory response of human aortic endothelial cells [51], forming a signal loop for regulating inflammatory responses in cardiovascular systems. BMAL1 and REV-ERBα can also regulate inflammatory cytokines, like chemokine C-C Motif Chemokine Ligand 2 (CCL2) and interleukin-6 (IL-6), and affect the rhythmicity of macrophage chemotaxis [74]. Tumor necrosis factor-α (TNF-α) expression is increased in cardiac matrix fibroblasts and macrophages of cardiac-specific *BMAL1 knockout mice* [75]. The downregulation of the biological clock gene BMAL1/CLOCK leads to circadian rhythm disruption and significant elevation of the pro-inflammatory cytokines CCL2, Interleukin-1 Beta( IL-1β), and IL-6 at the site of myocardial infarction in mice fed a high-fat diet, triggering cardiac inflammation [76]. The upregulation of *PER2*, a downstream target gene of BMAL1, decreases the activation of mouse caspase-3 and pro-inflammatory cytokine production to protect the myocardium from ischemic injury [77]. The SCN in mammals is a circadian pacemaker, which coordinates internal physiological processes and environmental cycles. RNA sequencing was performed on cardiac tissue from bilaterally *SCN-ablated mice* after myocardial infarction, which identified differentially expressed genes related to inflammatory responses and cytokine interactions. The inhibition of SCN function could improve the inflammatory response after myocardial infarction and promote cardiac repair by upregulating BMAL1 to promote insulin-like growth factor 2 (Igf2) expression and inducing macrophage conversion to an anti-inflammatory phenotype [78].

The above studies suggest that chemokines and cytokines can control the migration patterns of immune cells and the generation and regulation of the body’s inflammatory immune response. BMAL1 can rhythmically modulate the inflammatory response through the regulation of inflammation-associated cytokines and chemokines and ameliorate the adverse consequences of myocardial ischemic infarction.

### 4.4. BMAL1 Is Involved in Apoptosis and Autophagy Regulation

**Figure 1:**
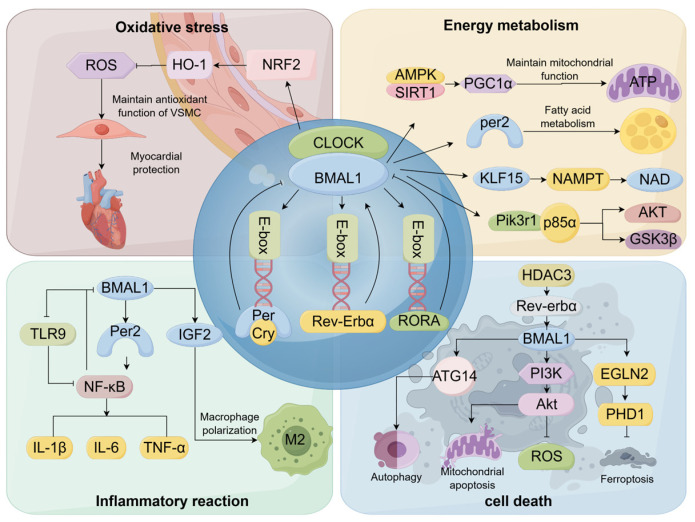
Regulatory mechanisms of myocardial ischemia by BMAL1. By Figdraw. BMAL1 (Brain and Muscle ARNT-like 1); CLOCK (Circadian Locomotor Output Cycles Kaput); Per (Period); Cry (Cryptochrome); Rev-Erbα (Nuclear Receptor Subfamily 1 Group D Member 1, NR1D1); RORA (Retinoic Acid Receptor-Related Orphan Receptor Alpha); ROS (Reactive Oxygen Species); HO-1 (Heme Oxygenase-1); NRF2 (Nuclear Factor Erythroid 2-Related Factor 2); AMPK (AMP-activated Protein Kinase); SIRT1 (Silent Information Regulator 1); PGC1α (Peroxisome Proliferator-Activated Receptor Gamma Coactivator 1-alpha); ATP (Adenosine Triphosphate); Per2 (period circadian regulator 2); KLF15 (Krüppel-like Factor 15); NAMPT (Nicotinamide Phosphoribosyltransferase); NAD (Nicotinamide Adenine Dinucleotide); Pi3kr1 p85α (Phosphoinositide-3-Kinase Regulatory Subunit 1 p85α); AKT (Protein Kinase B); GSK3β (Glycogen Synthase Kinase 3 Beta); TLR9 (Toll-like Receptor 9); NF-kB (Nuclear Factor Kappa-Light-Chain-Enhancer of Activated B Cells); IGF2 (Insulin-like Growth Factor 2); TNF-α (Tumor Necrosis Factor Alpha); IL-1β (Interleukin-1 Beta); IL-6 (Interleukin-6); HDAC3 (Histone Deacetylase 3); ATG14 (Autophagy Related 14); PI3K (Phosphatidylinositol 3-Kinase); Akt (Protein Kinase B); EGLN2 (EGL homolog 2); PHD1 (Prolyl Hydroxylase Domain-containing Protein 1).

Cell death is a significant risk factor contributing to the unfavorable prognosis of myocardial infarction, and cardiomyocyte death programs include but are not limited to autophagy, apoptosis, ferroptosis, and pyroptosis. Autophagy is a cellular process involving the generation of autophagosomes, which sequester cytoplasmic materials such as organelles, proteins, and metabolic byproducts requiring disposal. These double-membrane vesicles subsequently merge with lysosomes to create autolysosomes, enabling the enzymatic breakdown of their encapsulated contents through hydrolytic digestion. As a biological process of cellular metabolism and organelle renewal, autophagy is involved in the regulation of a variety of cardiovascular diseases, and overactivation leads to a reduction in the ability of cardiomyocytes to adapt to toxic environments [79]. The volume and number of autophagic vesicles in the heart fluctuate according to the circadian rhythm, peaking in the evening and then beginning to decline, suggesting that the biological process of autophagy is closely related to the biological clock gene [80]. The expression levels of autophagy-related 14 (*Atg14*), an important gene for the initiation of autophagy, also follow a circadian rhythm and are managed by a core clock component, the BMAL1/CLOCK complex [81]. In the rat model, the dysregulation of *BMAL1* gene oscillations induces mitochondrial autophagy dysfunction, which triggers cellular autophagy. The molecular mechanism is closely related to Histone Deacetylase 3 (HDAC3), a crucial element of the circadian negative feedback loop, which upregulates BMAL1 to restore mitochondrial autophagy by regulating the REV-ERBα/BMAL1 pathway, thereby alleviating myocardial infarction injury [82]. Moreover, cell experiments show that BMAL1 maintains the circadian rhythm of ischemic cardiomyocytes and reduces cell apoptosis, thereby protecting myocardial cell survival by regulating the PI3K/AKT signaling pathway and oxidative stress levels [83]. Ferroptosis is a type of programmed cell death triggered by the buildup of iron and lipid peroxidation. An overload of iron causes mitochondria to produce reactive oxygen species (ROS), resulting in the buildup of lipid peroxide that directly or indirectly damages macromolecular proteins, nucleic acids, and lipids, triggering cellular damage and death [84]. Research has indicated that iron homeostasis has a circadian rhythm [85]. Reduced BMAL1 in mice promotes ferroptosis by mediating oxidative damage via the EGL homolog 2/ Prolyl Hydroxylase Domain-containing Protein 1 (EGLN2/PHD1) [86](Figure 1).

The above studies indicate that cardiomyocyte death is closely related to BMAL1, which is involved in regulating the expression of cardiomyocyte death-related proteins, and that understanding the intrinsic molecular mechanisms of cardiomyocyte death may reduce the degree of myocardial injury after myocardial ischemia and improve its prognosis (Table 1).

## 5. Discussion

**Figure 2:**
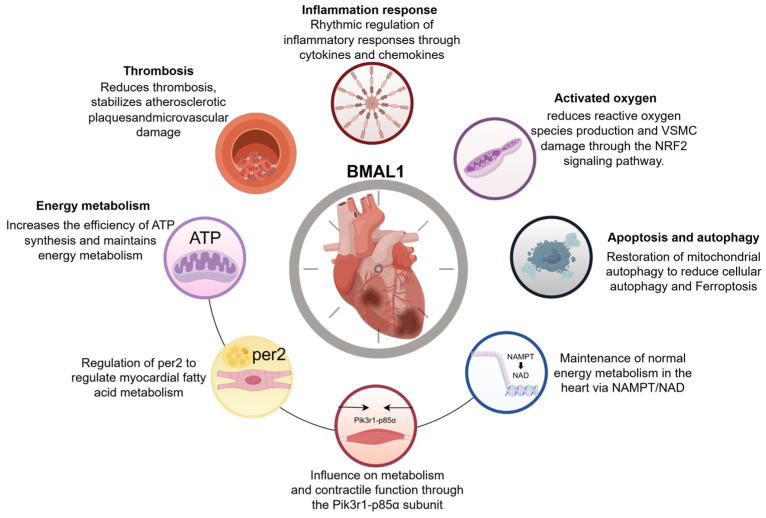
BMAL1’s regulatory role in the heart. By Figdraw.

The multifaceted regulatory roles of BMAL1 in cardiac health and disease underscore the critical importance of circadian clocks in maintaining circadian rhythm homeostasis to ensure physiological stability and preserve organ structural and functional integrity. By regulating the oxidative stress, BMAL1 helps protect cardiomyocytes from reactive oxygen species damage while maintaining cellular antioxidant capacity through the activation of antioxidant pathways, such as the NRF2 signaling pathway. In terms of energy metabolism, BMAL1 maintains cardiac energy supply and metabolic homeostasis by affecting mitochondrial function and regulating the expression of key metabolic genes. In addition, BMAL1 may influence the cardiac remodeling and repair process after myocardial infarction by regulating inflammatory mediators. The regulation of apoptosis and autophagy further revealed the role of BMAL1 in cardiac diseases. BMAL1 regulates cardiomyocyte survival and death by affecting autophagy-related gene expression and ferroptosis-related pathways. These results not only contribute to understanding the pathogenesis of heart disease but also offer potential targets for future therapeutic strategies(Figure 2).

With further research on BMAL1 and its associated pathways, we hope to develop new therapeutic tools to regulate the biological clock, improve cardiac metabolism, reduce inflammatory responses, and, ultimately, improve the prognosis of heart disease. Future research needs to focus on the specific mechanisms of action of BMAL1 and how to treat heart disease by interfering with these mechanisms. For example, one study used CRISPR/Cas9-mediated gene editing technology to generate *BMAL1-deficient human stem cells* and a non-human primate research model, through which the application of the model revealed that the core rhythmic protein BMAL1 has the novel function of maintaining genome stability, inhibiting transposon LINE1 activation, and antagonizing the aging of primate tissues and cells [87], which provides a new model and new technology for the mechanism of BMAL1 research and provides new models and techniques that may be applied to myocardial ischemia research in the future. A meta-analysis of clinical trials shows that 75% of patients exhibit differences in drug efficacy or toxicity depending on the timing of drug administration [88]. Chronotherapy is a biological clock-based dosing strategy that optimizes therapeutic effects by giving medications at specific times of the day. A prime example is that asthmatics are prone to nocturnal exacerbations due to nocturnal recruitment of inflammatory cells into the airways; therefore, prednisone administered at 3 pm is more effective in counteracting the inflammatory milieu and decreased lung capacity related to nocturnal exacerbations in asthma compared with other times during the day [89]. Thus, circadian rhythms are important for drug development and clinical therapy, contributing to more precise timing of drug administration, better pharmacokinetic properties, improved drug efficacy, and reduced toxicity associated with drug metabolism. For instance, based on the effect of BMAL1 on atrial fibrillation, as mentioned above, BMAL1 may play a role in inflammatory regulation, electrical remodeling, structural remodeling, and time therapy in patients with atrial fibrillation after PCI. In the future, it is necessary to further explore the BMAL1 gene editing technology and the clinical application of circadian rhythm-targeted drugs in order to realize the individualized antithrombotic strategy of “treating according to the time” [90]. Given the apparent circadian rhythmicity of cardiovascular disease, studies targeting small molecule modulators of BMAL1 may have an important role in the treatment of myocardial ischemia, with a view to bringing about more personalized and effective therapeutic regimens for patients with cardiovascular disease.

There are also some limitations in the research on the regulatory role of BMAL1 in heart diseases. At the mechanism level, its interaction with circadian rhythm networks (such as CLOCK and PER/CRY) and non-rhythm factors has not been fully clarified, and the existing literature retrieval may lead to incomplete mechanism analyses. In the aspect of clinical transformation, due to the lack of clinical experimental data, we cannot analyze whether BMAL1 levels or polymorphism can predict human clinical results through multivariate or regression models. In addition, animal/cell models are difficult to simulate the complexity of human pathology and may overestimate the transformation potential of preclinical research. Moreover, there may be some problems, such as insufficient statistical robustness, including small sample size, unreasonable design of control groups, and uncorrected *p*-values in multiple comparisons, which may affect the credibility of the conclusion. This complexity is particularly evident in circadian rhythm research involving animal models, where multi-parameter measurements and time-series sampling across different zeitgeber periods inherently generate numerous *p*-values. To prioritize narrative clarity and avoid overwhelming readers with extensive statistical data, we have chosen not to present all *p*-values in detail within the main text. Furthermore, the safety and ethical risks of new interventions, such as gene editing or small molecule regulation, still need to be systematically evaluated. In the future, it is necessary to integrate multidisciplinary evidence and standardize retrieval strategies to promote the clinical transformation of circadian rhythm-targeted therapy.

## 6. Conclusions

In summary, the multifaceted role of BMAL1 in cardiac homeostasis and disease pathogenesis highlights the central position of the circadian clock system in cardiovascular physiology and pathology. BMAL1 dysfunction disrupts cardiovascular homeostasis and accelerates injury progression, while its functional restoration holds therapeutic potential. As the underlying mechanisms become further elucidated, BMAL1 may emerge as a critical therapeutic target for ischemic heart disease. Future investigations can employ multivariate regression models to validate whether BMAL1 expression levels or genetic polymorphisms can serve as predictive biomarkers for clinical prognosis. Crucially, therapeutic strategies leverage BMAL1’s circadian properties, such as chronotherapy and tissue-targeted gene modulation. By integrating chronobiology with cardiology, BMAL1-centered interventions could redefine the management paradigm for ischemic heart disease, transforming chronobiological concepts from theoretical frameworks into clinical tools for personalized medicine. These advancements promise new therapeutic horizons for cardiac patients and may improve cardiovascular disease outcomes.

## Figures and Tables

**Table 1:** Molecular mechanisms and effects of BMAL1 in myocardial ischemia. IHD(Ischemic heart disease); BMAL1 KO(Bmal1 gene knockout); H9C2 (Rat Embryonic Cardiomyocyte Cell Line H9C2); I/R(Ischemia/Reperfusion);SCN(suprachiasmatic nucleus); MI (Myocardial in-farction).

	Model	Species	Target/Pathway	Molecular Mechanism	Observed Effect on IHD	Reference(s)
Oxidative Stress	Cardiac function decline model	Mouse (BMAL1 KO)	ROS clearance	BMAL1 deficiency leads to ROS accumulation; antioxidant therapy partially rescues cardiac function.	Affect cardiac function and myocardial senescence.	[49]
	Atherosclerosis model	Mouse (BMAL1 KO)	ROS/NF-κB	BMAL1 downregulation enhances TLR-NF-κB signaling, increasing ROS and monocyte recruitment.	Accelerated atherosclerosis, elevated IHD risk.	[50,51]
	H9C2 cardiomyocyte injury model	H9C2 rat cardiomyocytes	NRF2/HO-1	BMAL1 overexpression activates NRF2/HO-1, reducing ROS and apoptosis.	Attenuated oxidative damage.	[52]
	Diabetic rat I/R model	Rat	NRF2/HO-1	BMAL1 activates NRF2/HO-1 to reduce oxidative stress and lysosomal dysfunction.	Reduced susceptibility to ischemia–reperfusion injury.	[55]
Energy Metabolism	Heart disease model	Mouse	SIRT1/PGC1α, CLOCK/BMAL1-ROS/mitochondrial fission	BMAL1 damages mitochondrial function through the SIRT1-PGC1α axis, while CLOCK/BMAL1 interactions reduce ROS, inhibit division, enhance electron transfer, and alleviate oxidative stress.	BMAL1 deficiency causes mitochondrial dysfunction and impaired ATP synthesis. Insufficient myocardial energy supply.	[60,61,62,63]
	Ischemia and reperfusion model	Mouse (PER2 KO)	PER2/Fatty acid metabolism	BMAL1 regulates PER2 via the CLOCK/BMAL1 heterodimer binding to E-Box, activating PER2 transcription.	PER2 deletion impairs glycolysis, depletes glycogen stores, and increases infarct size.	[65,66]
	Cardiomyocyte-specific BMAL1 knockout model	Mouse (BMAL1 KO)	PIK3R1-p85α (PI3K/AKT/GSK3β)	BMAL1 regulates the circadian expression of PIK3R1, a key insulin signaling component.	Impaired insulin signaling cascade, leading to metabolic and contractile dysfunction.	[67]
	Ischemia and reperfusion model	Mouse	NAMPT/NAD	BMAL1/CLOCK-KLF15 axis regulates circadian NAMPT expression; KLF15 KO disrupts NAD rhythm.	NAD deficiency causes energy stagnation and abnormal cardiac metabolism.	[68,69,70]
Immune-Inflammatory Response	MI	Mouse	BMAL1/Neutrophil aging	BMAL1 regulates circadian oscillations of neutrophils; loss of BMAL1 disrupts neutrophil aging and infiltration.	Reduced angiogenesis, increased fibrosis, and impaired cardiac remodeling post-MI.	[43,72]
	Atherosclerosis model	Mouse, human aortic endothelial cells	TLRs-NF-κB/DNMT-1	TLRs-NF-κB axis inhibits BMAL1 via DNMT-1-mediated promoter methylation; BMAL1 loss enhances NF-κB signaling.	Elevated neutrophil chemotaxis, pro-inflammatory gene expression, and cardiac fibrosis.	[51]
	Cardiomyocyte-specific BMAL1 knockout model	Mouse (BMAL1 KO)	TNF-α/SMAD3	BMAL1 deletion elevates TNF-α expression in cardiac fibroblasts and macrophages.	Enhanced myocardial fibrosis and inflammatory signaling.	[75]
	High-fat diet + circadian disruption + MI	Mouse	BMAL1/Clock/Pro-inflammatory cytokines	BMAL1/CLOCK downregulation increases CCL2, IL-1β, and IL-6 at MI sites.	Exacerbated cardiac inflammation and adverse remodeling under metabolic stress.	[76]
	SCN-ablated MI model	Mouse	BMAL1/Igf2/Macrophage phenotype	SCN ablation upregulates BMAL1, promoting Igf2 expression and anti-inflammatory macrophage polarization.	Improved post-MI inflammation resolution and cardiac repair.	[78]
Apoptosis and Autophagy	Diabetic rats with I/R injury	Rat	HDAC3/REV-ERBα/BMAL1 pathway	HDAC3 upregulates BMAL1 to restore mitochondrial autophagy via REV-ERBα/BMAL1 signaling.	Alleviates myocardial infarction injury.	[82]
	Myocardial ischemia model	H9C2 rat cardiomyocytes	PI3K/AKT signaling and oxidative stress	BMAL1 maintains the circadian rhythm of ischemic cardiomyocytes by regulating PI3K/AKT and oxidative stress.	Reduces apoptosis, protects cardiomyocyte survival.	[83]
	Immunodeficient mice inoculated with human tumor cells	Mouse	EGLN2/PHD1	Reduced BMAL1 promotes ferroptosis via EGLN2/PHD1-mediated oxidative damage.	Aggravates ferroptosis-related cell death.	[85,86]

## Data Availability

This review article is based on previously published studies. All data analyzed in this work are publicly available through the cited references. No new datasets were generated or analyzed during this review. Links to the original sources are provided in the reference list.
